# Characterization of IL-22 Bioactivity and IL-22-Positive Cells in Grass Carp *Ctenopharyngodon idella*

**DOI:** 10.3389/fimmu.2020.586889

**Published:** 2020-10-06

**Authors:** Yibin Yang, Junya Wang, Jiawen Xu, Qin Liu, Zixuan Wang, Xiaozhen Zhu, Xiaohui Ai, Qian Gao, Xinhua Chen, Jun Zou

**Affiliations:** ^1^Key Laboratory of Exploration and Utilization of Aquatic Genetic Resources, Ministry of Education, Shanghai Ocean University, Shanghai, China; ^2^International Research Center for Marine Biosciences at Shanghai Ocean University, Ministry of Science and Technology, Shanghai, China; ^3^National Demonstration Center for Experimental Fisheries Science Education, Shanghai Ocean University, Shanghai, China; ^4^Yangtze River Fisheries Research Institute, Chinese Academy of Fishery Sciences, Wuhan, China; ^5^Key Laboratory of Marine Biotechnology of Fujian Province, Institute of Oceanology, Fujian Agriculture and Forestry University, Fuzhou, China; ^6^Laboratory for Marine Biology and Biotechnology, Qingdao National Laboratory for Marine Science and Technology, Qingdao, China

**Keywords:** interleukin 22, IL-22 producing cells, cytokine, bioactivity, grass carp

## Abstract

Interleukin (IL)-22 plays an important role in regulating inflammation and clearance of infectious pathogens. IL-22 homologs have been discovered in fish, but the functions and sources of IL-22 have not been fully characterized. In this study, an IL-22 homolog was identified in grass carp and its bioactivities were investigated. The grass carp IL-22 was constitutively expressed in tissues, with the highest expression detected in the gills and hindgut. It was upregulated in the spleen after infection with *Flavobacterium columnare* and grass carp reovirus and in the primary head kidney and spleen leukocytes stimulated with LPS and IL-34. Conversely, it was downregulated by Th2 cytokines such as IL-4/13B and IL-10. The recombinant IL-22 produced in bacteria showed a stimulatory effect on the expression of inflammatory cytokines and STAT3 in the primary head kidney leukocytes and CIK cells. Moreover, the IL-22-positive cells were found to be induced in the hindgut and head kidney 24 h after infection by *F. columnare*. Our data suggest that IL-22 plays an important role in regulating mucosal and systemic immunity against bacterial and viral infection.

## Highlights

- An IL-22 homolog was identified in grass carp.
- IL-22 is upregulated during infection of *Flavobacterium columnare* and grass carp reovirus.
- IL-22 is induced in primary leukocytes by IL-34 but downregulated by IL-4/13B and IL-10.
- IL-22 upregulates inflammatory cytokines and STAT3.
- The IL-22-positive cells are increased in number in the hindgut and head kidney after infection by *F. columnare*.

## Introduction

Interleukin (IL)-22 belongs to the IL-10 cytokine family and consists of 6 alpha helices. It is mainly produced by activated Th17 cells, natural killer (NK) cells, and innate lymphoid cells (ILCs) and acts upon a wide range of cell types such as T cells, macrophages, epithelial cells, stem cells, fibroblasts, and keratinocytes (1, 2). IL-22 acts to generate chemokines, inflammatory factors, and antimicrobial peptides (AMPs) (3, 4) and mediates mucosal defenses to subsequently limit bacterial replication and facilitate pathogen clearance by promoting the production and secretion of AMPs, by enhancing the phagocytic activities of innate cells, and by inhibiting the autophagous processes of target cells (5). Excessive levels of IL-22 can cause malfunctioning of the immune system, resulting in a failure to clear pathogens and in chronic inflammatory diseases such as psoriasis (6).

IL-22 binds to a heterodimeric receptor complex consisting of a high binding affinity chain (IL-22R1) and IL-10R2, which are also shared by IL-10, IL-19, IL-20, IL-24, and IL-26 (7). Cellular signaling of IL-22 mainly involves activation of Janus kinase/signal transducers together with the activators of the transcription (JAK/STAT) signaling pathway, the p38 pathway, the extracellular signal-regulated kinase/extracellular regulatory protein kinase pathway, and the JNK/SAPK pathway (8). The functions of IL-22 can be antagonized by use of a soluble IL-22 binding protein (IL-22BP) which shares high sequence homology with IL-22R1 and thus competes with IL-22R1 for the binding site in IL-22 (9, 10).

The IL-22 gene is present in all jawed vertebrates and is clustered with the IFN-γ gene in the genome. Across species, most IL-22 genes are organized into 6 exons and 5 introns. Exceptions include the IL-22 genes in haddock (*Melanogrammus aeglefinus*) and pufferfish (*Fugu rubripes*) which are comprised of 5 exons and 4 introns. Fish IL-22 proteins span 170–190 amino acids (aa) with a hydrophobic signal peptide of 25–30 aa and are structurally conserved. The crystal structure of zebrafish (*Danio rerio*) IL-22 has been assessed and determined to display a typical class II cytokine structure comprised of 6 α helices (11). Fish IL-22 has been found to be highly expressed in mucosal tissues such as the intestine and gills, and its production can be induced in response to proinflammatory stimuli such as LPS and cytokines (12–17). For example, LPS-based treatments of intestinal cells that were freshly isolated from Mandarin fish (*Siniperca chuatsi*) resulted in 10–20-fold increases in IL-22 transcription levels (17). Proinflammatory cytokines such as IL-1β and TNF-α are also potent inducers of IL-22 expression (18, 19). Further, it was found that IL-22 was markedly upregulated at mucosal sites in fish infected with gram-negative or gram-positive bacterial pathogens (15). Recently, IL-22-producing cells were characterized in rainbow trout (*Oncorhynchus mykiss*) and were found to reside in gill filaments and interbranchial lymphoid tissues, having accumulated thereafter the fish infected with *Aeromonas salmonicida* (20). That study demonstrated that IL-22 is a key regulator, which coordinates the immune responses against bacterial pathogens (20).

IL-22 bioactivity has been examined in several fish species, including Mandarin fish (17), pufferfish (21), zebrafish (13), rainbow trout (12), So-iuy mullet (*Liza haematocheila*) (22), turbot (*Scophthalmus maximus*) (14), Atlantic cod (*Gadus morhua*), and haddock (23). IL-22 has been shown to induce the expression of antimicrobial peptide genes including defensins, hepcidin, and LEAP-2 in the primary leukocytes isolated from the spleen and intestine tissues (12, 17). This suggests that IL-22 is essential for the initiation of anti-bacterial defenses in fish. As in mammals, the IL-22-activated antimicrobial responses in fish can be antagonized by the IL-22-binding protein (18). *In vivo* administration of recombinant IL-22 indeed enhanced protection of *L. haematocheila* against *Streptococcus dysgalactiae* and *of S. maximus* against *Aeromonas hydrophila* (14, 22). The studies highlight the central role and importance of IL-22 in the dynamics and mechanisms underlying antibacterial immunity.

In this study, an IL-22 homolog was identified in grass carp (*Ctenopharyngodon idella, Ci*) and expression analyzed in response to PAMPs, cytokines, and bacterial and viral infection. The biological activities of recombinant IL-22 were examined in the modulation of immune genes in different cell types. In addition, monoclonal antibodies were produced against the recombinant IL-22 and characterized for detecting the IL-22-producing cells.

## Materials and Methods

### Fish

Grass carp (*Ctenopharyngodon idella*) (120 ± 10 g) were obtained from Binhai Base, Shanghai Ocean University, China. Fish were placed in tanks with aeration for at least 10 days before experimental procedures including intraperitoneal (i.p.) injection and tissue sampling. All experiments were conducted under the national regulations on use of laboratory animals of China and approved by the ethics committee of laboratory animals of Shanghai Ocean University (SHOU-DW-2019-003).

### Cloning and Identification of CiIL-22

Total RNA was extracted from spleen and kidney of healthy grass carp using the TRIzol Reagent (Invitrogen) according to the manufacturer's instructions. cDNA was synthesized using a PrimeScript Gamma II 1st-strand cDNA Synthesis Kit (Takara). The synthesized cDNA was stored at −20°C for gene cloning. The partial cDNA sequence of *Ci*IL-22 was obtained from the whole-genome database of grass carp (http://www.ncgr.ac.cn/grasscarp/) (24). A rapid amplification of cDNA ends PCR kit (Life Technology) was used to amplify the full-length sequence of *Ci*IL-22 using the specific gene primers in Table 1.

**Table 1:** Primers used in this study.

**Primers**	**Sequence (5^**′**^-3^**′**^)**	**Application**
Fa	GCACATCTTGCATGCAGATGATC	3′-RACE
Fb	GATCTGCACAGGCTCGCACAAG	3′-RACE
Fc	TGCAGAACATGCGCAGGTCAAG	3′-RACE
Ra	GTCTTCCTTCTGTGCATGTTCAG	5′-RACE
Rb	TAGAGGTTGTTCCAGGTGACG	5′-RACE
Rc	GGGGCGCGGGCGCATGAGGTGC	5′-RACE
IL-22-F	CTCGTCTACGAGGAACATCAGTC	Verify the full length
IL-22-R	GCATGAAAGCACAGTTCCCATGCC	Verify the full length
IL-22-qF	CCGTACTGTAGCAACAGTGCAG	Real-time PCR
IL-22-qR	TCACATTCTTGCAGAGCAGGATTC	Real-time PCR
IL-34-qF	TCAACAGGGTATAAAGAGGGTT	Real-time PCR
IL-34-qR	ATCCAGTAATGACTTGGGTGTA	Real-time PCR
IL-6-qF	CAGCAGAATGGGGGAGTTATC	Real-time PCR
IL-6-qR	CTCGCAGAGTCTTGACATCCTT	Real-time PCR
IL-1β-qF	TCTCCTCGTCTGCTGGGTGT	Real-time PCR
IL-1β-qR	CAAGACCAGGTGAGGGGAAG	Real-time PCR
IL-8-qF	TCTACCCTCCTAGCCCTCACTG	Real-time PCR
IL-8-qR	TCATGGTGCTTTGTTGGCAAGGA	Real-time PCR
IL-10-qF	GCAACAGAACATCAATAGTCCTT	Real-time PCR
IL-10-qR	CACCCTTTTCCTTCATCTTTTCA	Real-time PCR
TGF-β1-qF	TTGGGACTTGTGCTCTAT	Real-time PCR
TGF-β1-qR	AGTTCTGCTGGGATGTTT	Real-time PCR
IL-21-qF	CCACCAACGATTTGAAGGACTGC	Real-time PCR
IL-21-qF	CTGGGCAACTTTTCCACAATGA	Real-time PCR
STAT3-F	GGCTCTATGGAATGAAGGGTA	Real-time PCR
STAT3-R	CAACTGACTGGATCTGGGTCT	Real-time PCR
EF-1α-qF	CAGCACAAACATGGGCTGGTTC	Real-time PCR
EF-1α-qR	ACGGGTACAGTTCCAATACCTCCA	Real-time PCR
UPM-Long	CTAATACGACTCACTATAGGGCAAGC AGTGGTATCAACGCAGAGT	3′-RACE
UPM-Short	CTAATACGACTCACTATAGGGC	3′-RACE
NUP	AAGCAGTGGTATCAACGCAGAGT	3′-RACE
APG	CCAGACTCGTGGCTGATGCA GGGGGGGGGGGGGGGG	5′-RACE
AP	CCAGACTCGTGGCTGATGCA	5′-RACE
T7	TAATACGACTCACTATAGGG	Plasmid verification
T7-tet	GCTAGTTATTGCTCAGCGG	Plasmid verification
pcDNA3.1-F	CTAGAGAACCCACTGCTTAC	Plasmid verification
pcDNA3.1-R	TAGAAGGCACAGTCGAGG	Plasmid verification

### Sequence Analysis of CiIL-22

The nucleotide sequence of *Ci*IL-22 was assembled and analyzed by DNAMAN 8.0. using ORFfinder listed on the NCBI website (https://www.ncbi.nlm.nih.gov/orfinder/). Protein and nucleic acid translation was performed by Primer Premier 5.0. BLASTN and BLASTP (http://www.ncbi.nlm.Nih.gov/BLAST/) were used to identify the homologous sequences. Homology of sequences was analyzed using the Cluster Omega (https://www.ebi.ac.uk/Tools/msa/clustalo/). Signal peptide was predicted using SignalP program (version 3.0) (http://www.cbs.dtu.dk/services/SignalP/), and multiple-sequence alignment was generated using GeneDoc software. A phylogenetic tree was constructed using the Neighbor Joining method and repeated for 10,000 times to obtain the bootstrap values.

### Tissue Expression Analysis of CiIL-22

Seven tissues including liver, spleen, head kidney, hindgut, skin, gill, and thymus were taken from six healthy grass carp. Total RNA was extracted using the TRIzol Reagent and reverse transcribed into cDNA using the premix 2 × Hifair™ II SuperMix plus Kit (Yeasen). The synthesized cDNA samples were stored at −20°C until use.

Quantitative real-time PCR (qPCR) was performed using the iTaq™ Universal SYBR® Green Supermix (Bio-Rad) and run on the LightCycler 96 Real Time PCR System (Roche) to analyze gene expression. The qPCR conditions are as follows: 1 cycle of 95°C for 30 s and 40 cycles of 95°C for 10 s, 60°C for 20 s, and 72°C for 20 s. A 10-fold dilution of plasmid DNA containing the target gene fragment was used to establish a standard curve for each gene to quantify the transcription levels. Elongation factor 1α (EF-1α) was used as an internal reference gene to normalize gene expression (25). The expression levels of each gene were calculated as arbitrary units which were normalized to that of EF-1α. Fold changes of expression were calculated by comparing the average expression levels of the experimental groups with that of corresponding control groups. The primers used for qPCR are described in Table 1.

### Bacterial Challenge

The *Flavobacterium columnare* G4 strain, provided by the State Key Laboratory of Freshwater Ecology and Biotechnology, Institute of Hydrobiology, Chinese Academy of Sciences (26), was used for the challenge experiment. Bacteria were cultured in Shieh medium at 28°C for 48 h with continuous shaking at 200 rpm/min to reach logarithmic growth phase, centrifuged at 4,200 × g for 4 min and resuspended in PBS buffer. Bacteria were adjusted to a concentration of 1 × 10^7^ CFU/ml.

In the bacterial challenge experiment, 50 healthy grass carp were randomly divided into two groups, 25 fish in each group. Fish were injected intraperitoneally (i.p.) with 1 μl bacteria suspension (1 × 10^7^ CFU/ml) per gram body weight or the same volume of PBS. Spleen, thymus, hindgut, gill, and head kidney were collected at 24, 48, and 72 h after injection, and homogenized in the TRIzol reagent for total RNA extraction. Total RNA was reverse transcribed into cDNA for expression analysis.

### Viral Infection

Grass carp reovirus virus (GCRV) II was provided by the Institute of Virology, Chinese Academy of Sciences. In the infection experiment, 50 healthy grass carp were randomly divided into two groups, 25 fish in each group. Fish were i.p. injected with 200 μl GCRV solution (resuspended in DMEM, 1 × 10^7^ TCID_50_/ml) or 200 μl DMEM. Head kidney, thymus, gill, spleen, and hindgut were sampled at days 1, 3, 7, and 14 and homogenized in the TRIzol reagent for total RNA extraction and cDNA synthesis.

### Production and Purification of Recombinant CiIL-22 in Bacteria

The predicted mature peptide of *Ci*IL-22 (starting from was M^21^) cloned into pET-21d (Invitrogen). The constructed plasmid was sequenced with T7 and T7-tet primers (Table 1) and transformed into *Escherichia coli* BL21 cells. The bacteria were induced with 1 mM IPTG to produce recombinant *Ci*IL-22 (r*Ci*IL-22) (27). r*Ci*IL-22 was expressed as inclusion bodies which were subject to denaturation, refolding, and purification using the Superdex 200 column (GE Healthcare). Purified r*Ci*IL-22 was analyzed by SDS-PAGE and concentration determined by the Bradford method. Protein was aliquoted and stored at −80°C.

### Modulation of CiIL-22 Expression in Head Kidney and Spleen Leukocytes and CIK Cells

Head kidney and spleen of grass carp were sampled for isolation of leukocytes as previously described (25, 28). Cells were counted and cultured in a 6-well plate at 10^6^ cells/well for 6 h. The cells were stimulated with different immune stimulants including poly(I:C) (50 μg/ml), PHA (10 μg/ml), and LPS (50 μg/ml), or cytokines including r*Ci*IL-2 (20 ng/ml), r*Ci*IL-4/13B (20 ng/ml), r*Ci*IL-10 (20 ng/ml), r*Ci*IL-34 (20 ng/ml), r*Ci*IFN-1 (20 ng/ml), or r*Ci*IFN-γrel (20 ng/ml). Poly(I:C), PHA and LPS were purchased from Sigma and recombinant cytokines purified in our laboratory. After stimulation, cells were collected for extraction of total RNA and gene expression analysis.

Head kidney leukocytes of grass carp were prepared by discontinuous density gradient centrifugation (25, 28). Isolated leukocytes were cultured on a 6-well plate for 6 h and stimulated with r*Ci*IL-22 for 12 h. The CIK cell line of grass carp was provided by the National Pathogen Collection Center for Aquatic Animals, Shanghai Ocean University. The CIK cells were seeded in 6-well plates and cultured for about 6 h and stimulated with r*Ci*IL-22. Total RNA was extracted from the cells for expression analysis of IL-1β, IL-6, IL-8, IL-10, IL-21, IL-22, IL-34, TGF-β1, and STAT3 by qPCR.

### Generation of CiIL-22 Monoclonal Antibody and Confocal Microscopy

The *Ci*IL-22 monoclonal antibody (mAb) was generated against the r*Ci*IL-22 by the Beijing Huada Protein Research and Development Center Co. Ltd. Western blotting was performed to verify the antibody specificity. Briefly, the r*Ci*IL-22 was separated by PAGE electrophoresis and transferred to the PVDF membrane using a semi-dry transfer method. The membrane was blocked with TBS buffer with 5% skimmed milk powder for 1 h and incubated with the primary antibody (diluted 1:1,000, v/v) with TBS containing 0.2% Tween 20 at room temperature for 1 h or at 4°C overnight. After washing with TBS-T (TBS containing 0.1% Tween 20) buffer for 3 × 5 min, the membrane was incubated with the goat anti-mouse IgG H & L (IRDye® 680RD, 1: 10,000 dilution, v/v, Odyssey) at room temperature for 1 h, washed with TBS-T (containing 0.1% Tween 20) buffer for 3 × 5 min, and photographed under the Odyssey CLx image system (Odyssey).

To validate the reactivity of mAbs with the r*Ci*IL-22 expressed in eukaryotic cells, the mature peptide of *Ci*IL-22 (starting from M^21^) was synthesized and inserted into pcDNA3.1 (Genewiz). The pcDNA3.1-*Ci*IL-22 plasmid was transfected into HEK293T cells and cultured at 37°C in a CO_2_ incubator for 48 h. The cells were lysed in RIPA buffer on ice for 10 min and used for Western blotting.

For confocal microscopy, the r*Ci*IL-22 mAb was labeled with FITC Fluor (HuaBio). Grass carp were infected with *F. columnare* as described above. Twenty-four hours post infection, the hindgut and kidney were fixed with 4% paraformaldehyde, dehydrated, and embedded (29), and cryo section was made. After antigen heat retrieval, the sections were incubated with *Ci*IL-22 monoclonal antibody overnight at 4°C, fully washed, and the nuclei were stained with DAPI (1 μg/ml, Beyotime Biotech, China). Fluorescent imaging was viewed on a confocal laser scanning microscope (Nikon, Japan) and analyzed with the NIS Elements Viewer Software (Nikon, Japan).

### Statistical Analysis

The qPCR data were analyzed using the SPSS package 20.0 (SPSS Inc., Chicago, IL, USA). One-way ANOVA and the LSD *post-hoc* test were used to determine the significance (^*^p < 0.05 or ^**^p < 0.01) between treatment group and control group.

## Results

### Sequence Identification of CiIL-22

**Figure 1:**
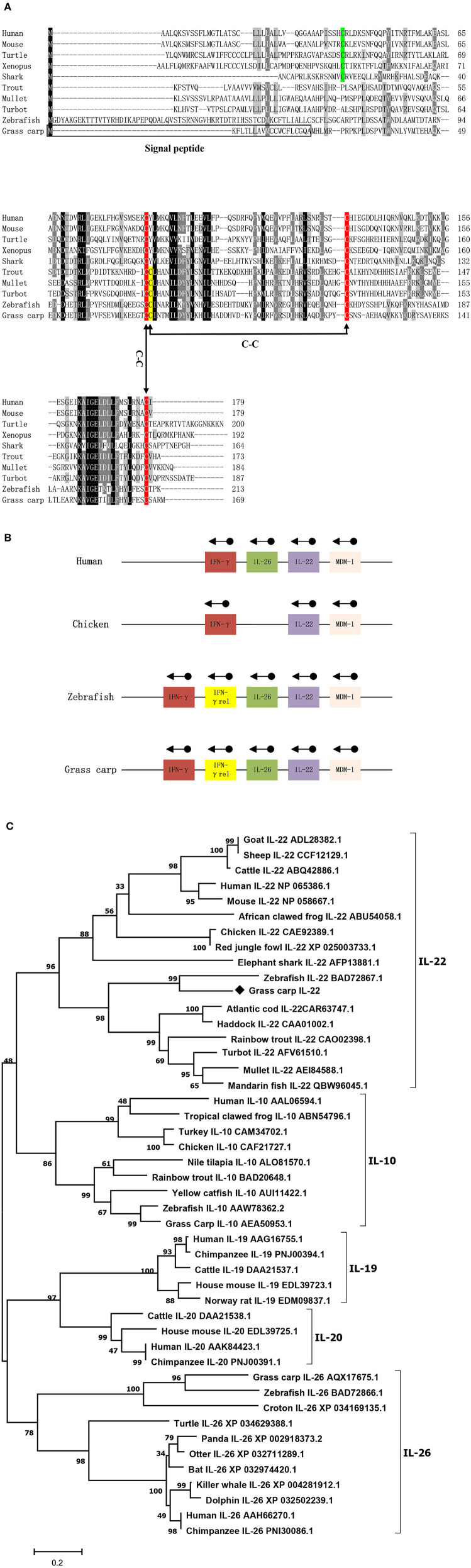
Protein sequence alignment **(A)**, gene synteny **(B)**, and the phylogenetic tree **(C)** of *Ci*IL-22 and IL-10 family members. The predicted signal peptide is boxed, and putative disulfide bonds of *Ci*IL-22 are indicated. Arrows indicate gene transcription orientation. The tree was constructed using the Neighbor-Joining method within the MEGA program (Version 6.0). The JTT matrix-based model using the pair-wise deletion option was chosen. The percentages of bootstrap values (>70%) are shown next to the branches based on 10,000 bootstrap replications. Protein sequences of IL-10 family members including IL-10, IL-19, IL-20, IL-22, and IL-26 were obtained from the NCBI database. The *Ci*IL-22 is indicated by “♦”.

The cDNA sequence of *Ci*IL-22 is 975 bp (NCBI accession number: MN643172) and contains an open reading frame (ORF) of 510 bp encoding a peptide of 169 aa (Figure 1). The *Ci*IL-22 gene consists of 5 exons and 4 introns and is located in the chromosomal locus containing IFN-γ and IL-26 (Figure 1B). Multiple-sequence alignment showed that the 4 cysteine residues forming 2 intramolecular disulfide bonds are conserved in fish (11) (Figure 1A). *Ci*IL-22 shares 21.2–54.1% sequence identity with known homologs (Table 2) and is placed in the IL-22 clade in the phylogenetic tree, supported by a branch bootstrap value of 96% (Figure 1C).

**Table 2:** Amino acid identities (%) of *Ci*IL-22 with other homologs.

	**1**	**2**	**3**	**4**	**5**	**6**	**7**	**8**	**9**
1. Human									
2. Mouse	77.65								
3. Turtle	45.51	42.13							
4. Frog	32.20	32.77	37.37						
5. Shark	31.13	30.46	29.01	25.62					
6. Turbot	20.13	18.87	16.47	15.98	17.93				
7. Grass carp	22.44	21.15	23.12	23.27	21.68	30.62			
8. Rainbow trout	21.43	21.43	18.47	17.31	22.46	48.82	28.03		
9. Zebrafish	20.51	19.87	25.95	20.75	18.57	29.34	54.09	47.06	
10. Mullet	17.39	16.77	16.27	14.37	18.44	59.89	30.00	24.22	26.63

### Analysis of CiIL-22 Expression in Fish

**Figure 2:**
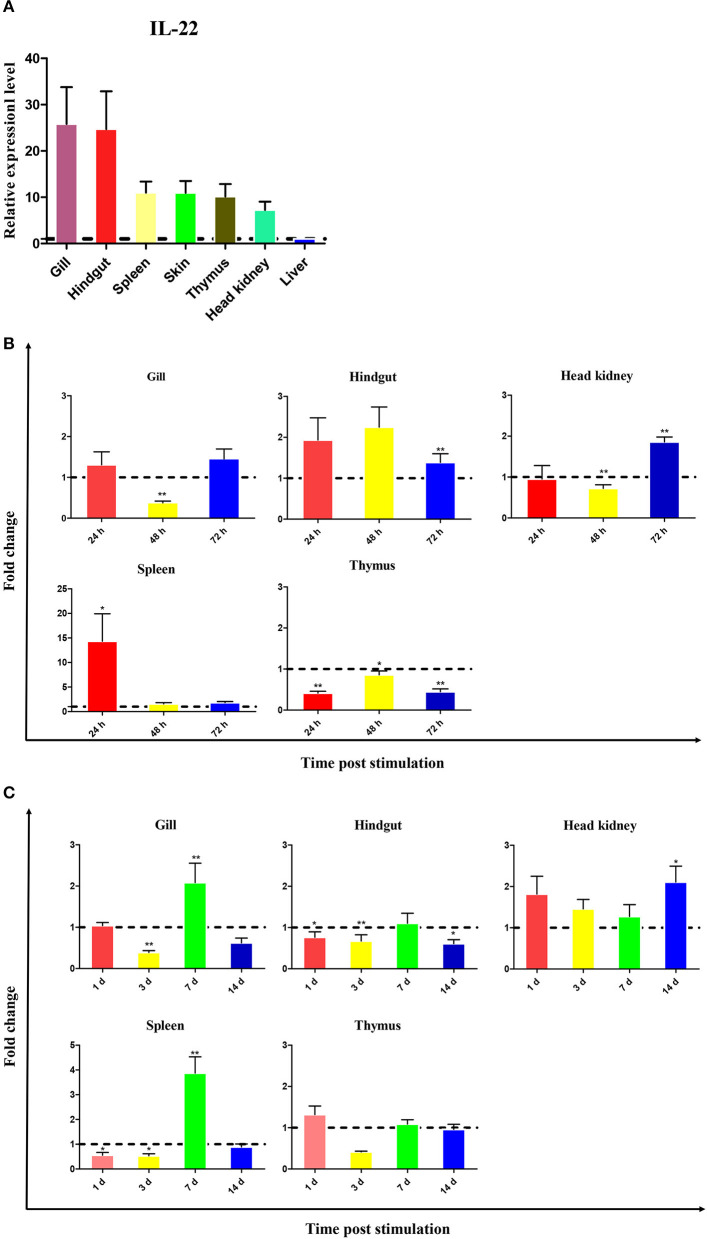
Expression of *Ci*IL-22 in tissues of healthy fish **(A)** and fish infected with *F. columnare* **(B)** or GCRV **(C)**. The mRNA levels of *Ci*IL-22 were determined by qPCR. The relative expression levels of *Ci*IL-22 were expressed as arbitrary units that were normalized against the expression levels of EF-1α. Fold changes were calculated by comparing the average levels of gene expression of infected groups with those of corresponding control groups. Data are shown as mean + SEM (*n* = 4). *p < 0.05 or **p < 0.01 are considered significant.

The expression of *Ci*IL-22 was examined in different tissues of healthy fish. These included the head kidney, liver, spleen, hindgut, skin, gills, and thymus. The expression levels of *Ci*IL-22 varied considerably among the tissues, with hindgut and gills displaying the highest levels of expression (Figure 2A).

To evaluate the IL-22 response to bacterial infection, fish were i.p. injected with *F. columnare*. Upregulation of *Ci*IL-22 expression was observed in the hindgut at 48 and 72 h post infection (hpi), in the head kidney at 72 hpi, and in the spleen at 24 hpi (Figure 2B). In contrast, *Ci*IL-22 expression was downregulated in the thymus at 24, 48, and 72 h hpi and in the head kidney at 48 hpi. *Ci*IL-22 expression was also investigated in fish during a 14-day challenge trial after GCRV infection. Induced expression was detected in the gills and spleen at day 7 and in the hindgut and head kidney at day 14 (Figure 2C).

### Modulation of CiIL-22 Expression in Primary Cells

**Figure 3:**
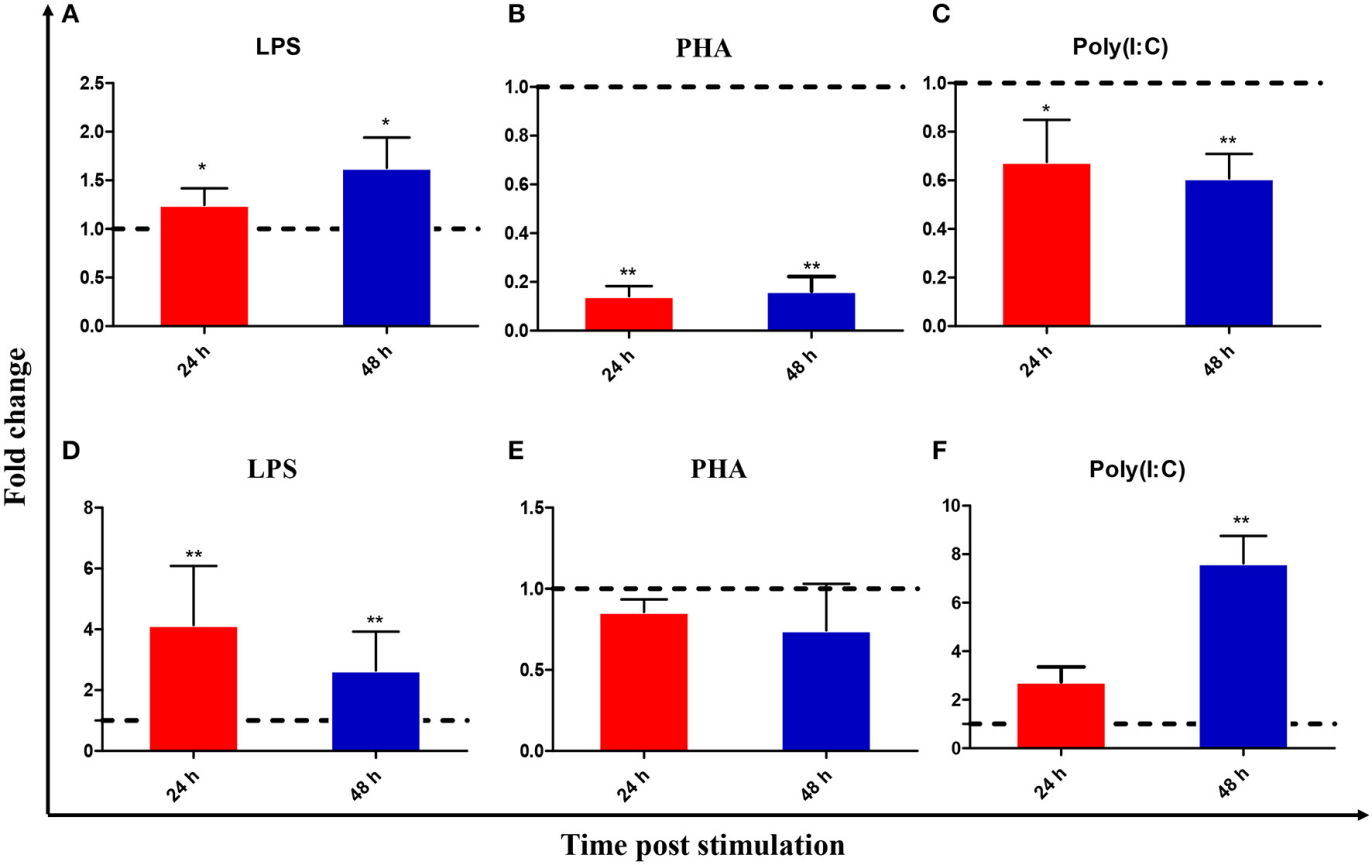
Expression analysis of *Ci*IL-22 in the primary head kidney **(A–C)** and spleen **(D–F)** leukocytes, after stimulation with LPS, PHA, or poly(I:C). The cells were stimulated with LPS, PHA or poly(I:C) for 24 and 48 h and analyzed by qPCR. Data are shown as mean + SEM (*N* = 4). *p < 0.05 or **p < 0.01 are considered significant.

**Figure 4:**
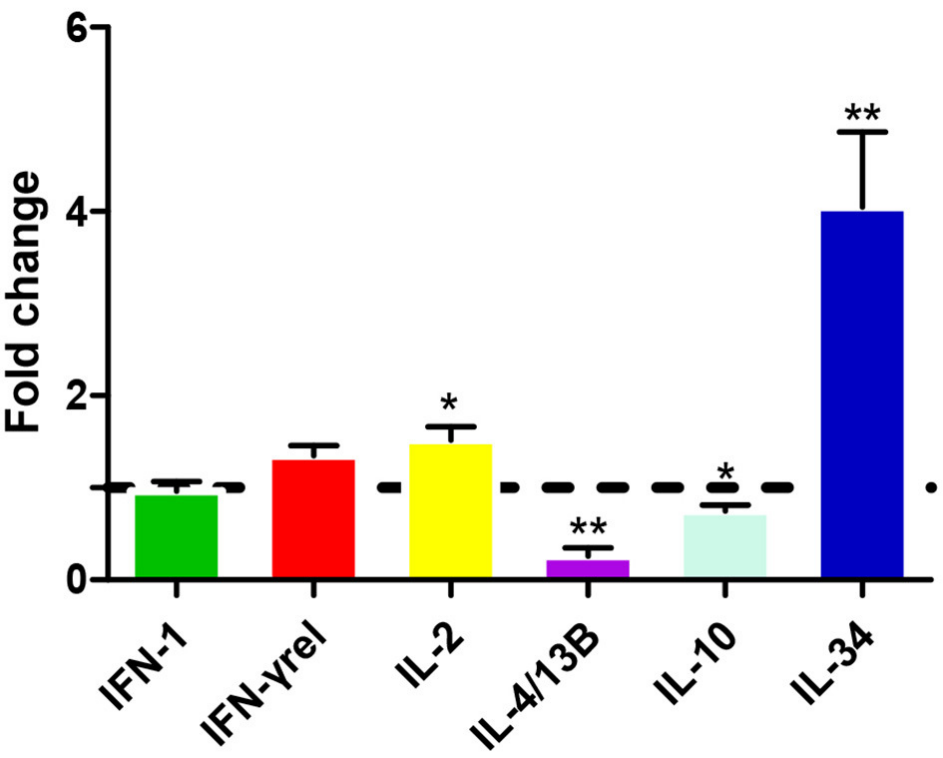
Expression analysis of *Ci*IL-22 in primary head kidney leukocytes after stimulation with r*Ci*IFN-1, r*Ci*IFN-γrel, r*Ci*IL-2, r*Ci*IL-4/13B, r*Ci*IL-10, or r*Ci*IL-34. Head kidney macrophages were stimulated with r*Ci*IFN-1, r*Ci*IFN-γrel, r*Ci*IL-2, r*Ci*IL-4/13B, r*Ci*IL-10, or r*Ci*IL-34 for 12 h, and *Ci*IL-22 expression was analyzed using qPCR. The EF-1α gene was used as an internal control. Fold change was calculated by comparing the average levels of expression of stimulated cells with those of corresponding control groups. Data are shown as mean + SEM (*N* = 4). *p < 0.05 or **p < 0.01 is considered significant.

Leukocytes were isolated from the head kidney and spleen and simulated with various stimuli to examine IL-22 expression. Figure 3 shows that *Ci*IL-22 expression was induced by LPS (50 μg/ml) in both tissues. PHA also decreased the expression of *Ci*IL-22 in the head kidney but had no effect in spleen leukocytes. 50 μg/ml of poly(I:C) had a stimulatory effect on the expression of *Ci*IL-22 in the spleen leukocytes at 48 h while it had an inhibitory effect in the head kidney leukocytes. PHA also decreased the expression of *Ci*IL-22 in the head kidney but had no effect in spleen leukocytes. To assess the effects of cytokines on the expression of *Ci*IL-22, primary head kidney leukocytes were incubated with 20 ng/ml of r*Ci*IL-2, r*Ci*IL-4/13B, r*Ci*IL-10, r*Ci*IL-34, r*Ci*IFN-1, or r*Ci*IFN-γrel for 12 h. *Ci*IL-22 was moderately upregulated by r*Ci*IL-2 and r*Ci*IL-34 but downregulated by r*Ci*IL-4/13B and r*Ci*IL-10 (Figure 4). r*Ci*IFN-1 and r*Ci*IFN-γrel had no effects on *Ci*IL-22 expression levels.

### Bioactivities of Recombinant CiIL-22 Protein

**Figure 5:**
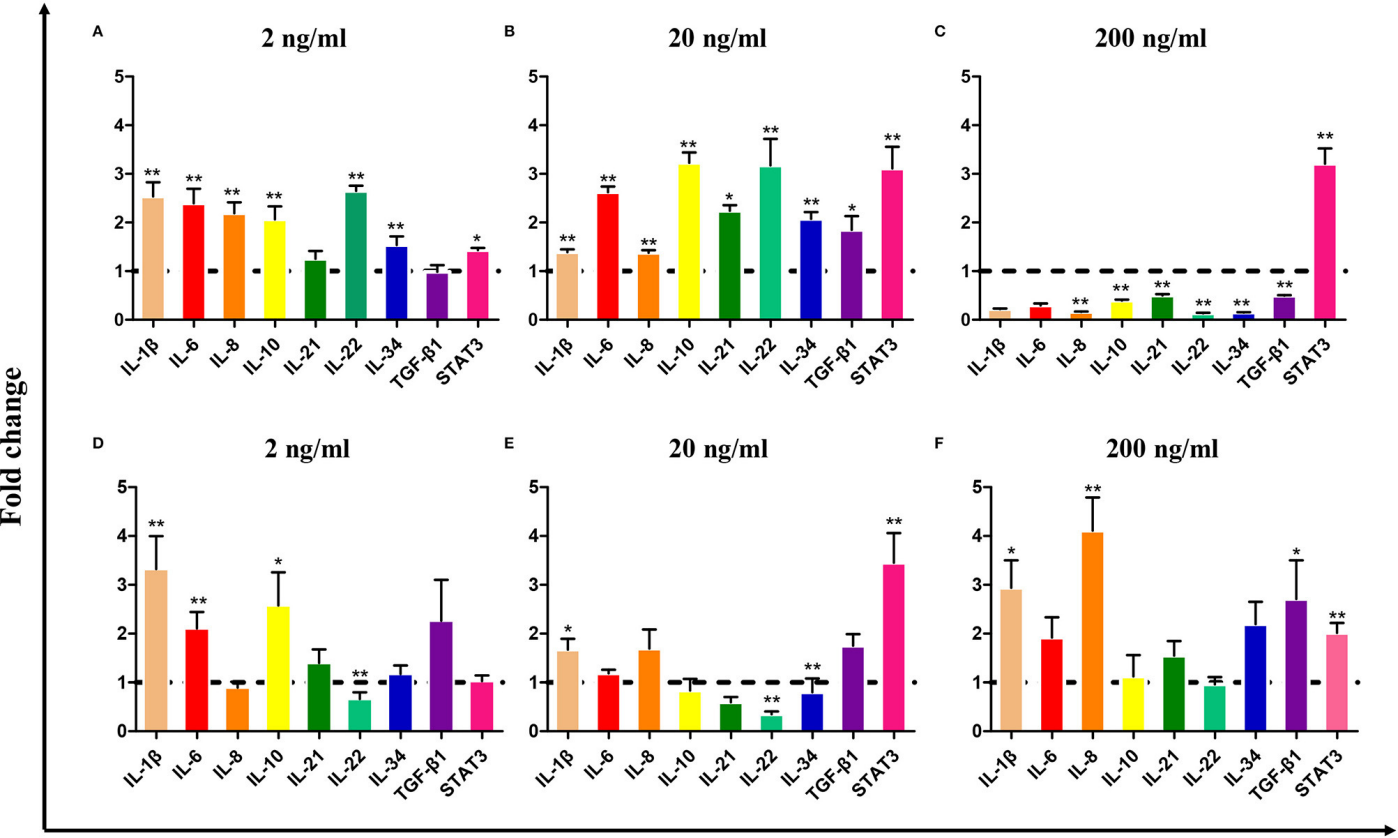
The effects of bacteria-derived r*Ci*IL-22 on gene expression in the primary head kidney leukocytes **(A–C)** and CIK cells **(D–F)**. The primary head kidney leukocytes were prepared as described in the Materials and Methods and stimulated with r*Ci*IL-22 for 12 h. The mRNA expression of cytokines and STAT3 was analyzed by qPCR. Data are shown as mean + SEM (*N* = 4). *p < 0.05 or **p < 0.01 is considered significant.

**Figure 6:**
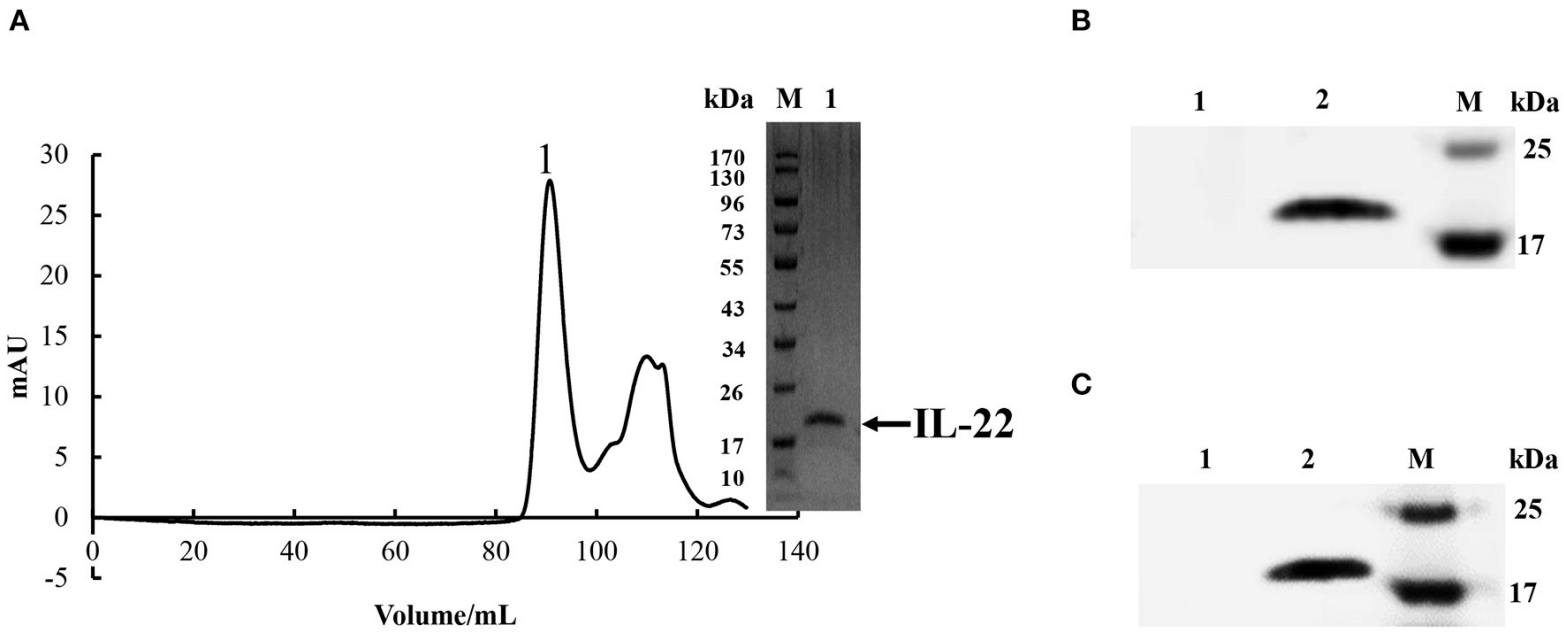
**(A)** Purification of r*Ci*IL-22 in bacteria. r*Ci*IL-22 was expressed in *E. coli* BL21 cells as inclusion bodies, refolded, and purified by size exclusion chromatography. Fraction 1 was checked by SDS-PAGE. **(B)** Western blotting analysis of bacteria-derived recombinant *Ci*IL-22 using an IL-22 monoclonal antibody (GC3-22). Lane 1: protein buffer; lane 2: purified r*Ci*IL-22. **(C)** Western blotting analysis of recombinant *Ci*IL-22 expressed in HEK293 cells using mAb GC3-22. Lane 1, cell lysate of HEK293 cells transfected with pcDNA3.1; lane 2, cell lysate of HEK293 cells transfected with pcDNA3.1-*Ci*IL-22.

To evaluate the biological activity of *Ci*IL-22, the recombinant *Ci*IL-22 (r*Ci*IL-22) protein was produced in bacteria and purified using size exclusion chromatography (**Figure 6**). Modulation of inflammatory cytokines by r*Ci*IL-22 was analyzed in the primary head kidney leukocytes and CIK cells. As shown in Figure 5, the expression of IL-1β, IL-6, IL-8, IL-10, IL-22, and IL-34 was induced by 2 and 20 ng/ml of protein, while 200 ng/ml of protein had inhibitory effects. However, the mRNA levels of IL-21 and TGF-β1 were only increased after stimulation with 20 ng/ml of *Ci*IL-22. In contrast, the CIK cells responded differently to r*Ci*IL-22 stimulation (Figure 5). Induced expression was detected for IL-1β (at all 3 doses), IL-8 (at 200 ng/ml), IL-10 (2 ng/ml), and TGF-β1 (200 ng/ml), while IL-22 expression was suppressed.

### Localization of IL-22-Producing Cells

**Figure 7:**
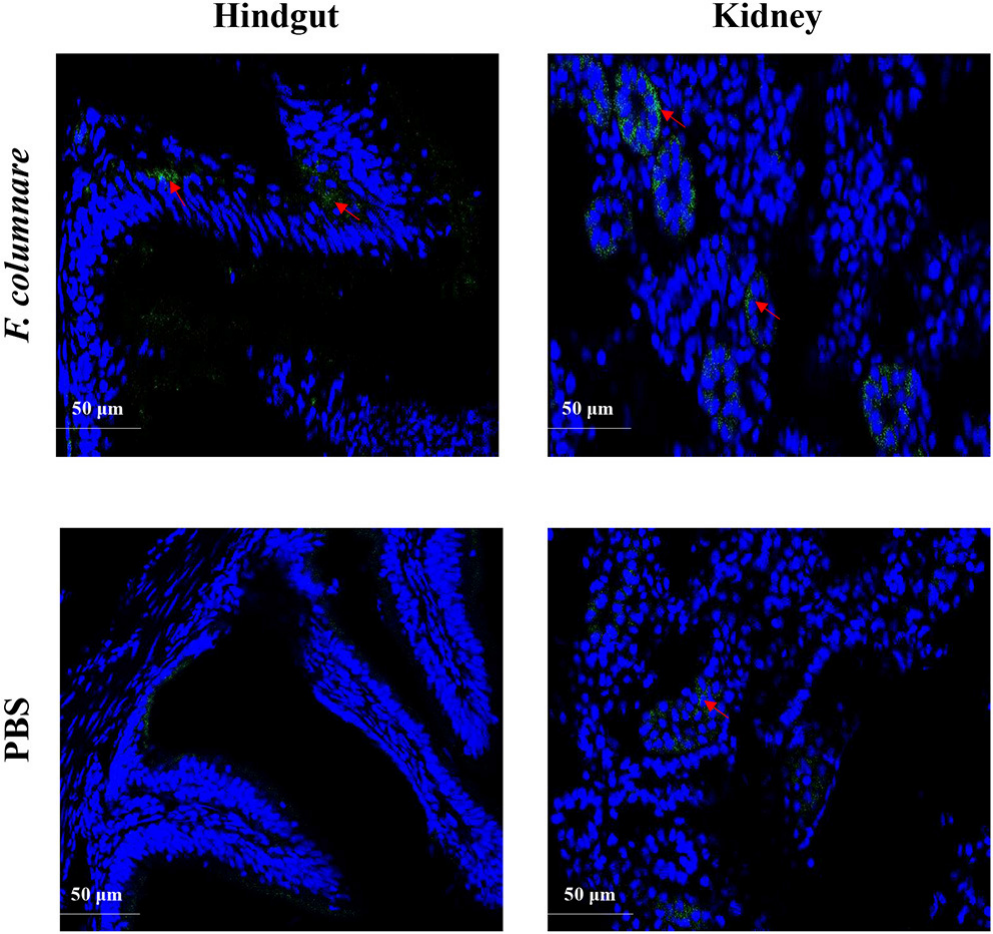
Localization of IL-22-positive cells in the hindgut and kidney of grass carp. Fish were i.p. injected with 1 μl bacterial suspension (1 × 10^7^ CFU/ml) per gram body weight or an equal volume of PBS. Hindgut and kidney were fixed at 24 h after injection and subjected to confocal microscopic analysis using a mAb (GC3-22) against *Ci*IL-22.

Monoclonal antibodies of *Ci*IL-22 were produced in mice using the purified r*Ci*IL-22 as an immunogen. Two positive clones were obtained, and their specificity was verified against the r*Ci*IL-22 using Western blotting. Clone GC3-22 was selected for further characterization using the cell lysate of HEK293 cells transfected with a plasmid expressing the mature peptide of *Ci*IL-22. As shown in Figure 6, a single protein of ~17.4 kDa was detected, confirming the specificity of *Ci*IL-22 mAb with the recombinant protein expressed in eukaryotic cells. To localize the IL-22-producing cells in fish infected with *F. columnare*, the mAb (GC3-22) was labeled with the FITC Fluor for confocal microscopy. As shown in Figure 7, clustered IL-22-positive cells were detected in the inner wall of the hindgut but not in PBS-injected fish. In the head kidney, weak staining was seen in the region surrounding the renal tubules in PBS-injected fish but markedly intensified in infected fish. Moreover, the numbers of stained tubules in infected fish were increased significantly.

## Discussion

In this study, an IL-22 homology was identified in grass carp. The IL-22 gene is present in all jawed vertebrates, mostly as a single gene. However, the IL-22 gene in polyploid fish exists as multiple copies and is clustered with the IFN-γ gene and IL-26 gene. This suggests that all these genes evolved from a common ancestor (12–15, 17, 22, 23). In teleost fish, this locus also contains an additional gene, termed IFN-γ rel, which has been duplicated from the IFN-γ gene (13, 30). The organization of the 5 exons and 4 introns separating the coding region of IL-22 also remains unaltered in all vertebrate species, including grass carp (Figure 1B) (23, 31). As for known IL-22 proteins, the translated *Ci*IL-22 protein was predicted to possess a signal peptide which can be cleaved to generate a mature peptide with a size comparable to IL-22s from other teleost fish species (13, 23). The *Ci*IL-22 of grass carp contained four cysteine (Cys) residues, three of which (Cys^73^, Cys^118^, and Cys^165^) were conserved in vertebrates. The fourth cysteine residue (corresponding to Cys^74^ in *Ci*IL-22) was aligned only within molecules derived from teleost fish (Figure 1A). Crystal structures of zebrafish IL-22 revealed that these four cysteines form 2 pairs of intramolecular disulfide bonds, namely Cys^73^-Cys^165^ and Cys^74^-Cys^118^. In humans, two disulfide bonds are also present (32). However, the positions of the cysteines forming the disulfide bonds in human IL-22 differ from the respective positions in fish IL-22. More specifically, the Cys^40^ of human IL-22 pairs with Cys^122^, and Cys^89^ pairs with Cys^178^. Intriguingly, the disulfide bonds seem to have little impact upon the overall protein topology of 6 α-helices (32).

IL-22 was constitutively expressed in most tissues with immune-related functions in healthy grass carp but was more highly expressed in the gills and hindgut (Figure 2). This observation is consistent with previous studies in which basal transcription was higher in mucosal tissues such as in the gills, pyloric cecae, and the intestine (12, 14, 17, 20, 23). These studies have suggested that IL-22 may mediate inflammation and facilitate the maintenance of homeostasis of the mucosal barrier in order to provide protection against pathogens. In a transgenic mouse-based modeling experiment, excessive expression of IL-22 resulted in both increased infiltration of macrophages into dermal layers and proliferation of keratinocytes, which caused subsequent thickening of the epidermis (33, 34). It has also been shown that IL-22 is involved in the inhibition of intestinal inflammation and tissue repair (3, 35, 36). For example, IL-22 promoted the healing intestinal trauma in assessments of acute intestinal injury (37) and also regulated intestinal flora under inflammation (38).

*In vitro* studies have indicated that *Ci*IL-22 is induced by LPS in primary leukocytes isolated from the head kidney and spleen (Figure 3). This is in line with previous studies where LPS was a robust inducer of IL-22 expression in cultured cells (8, 13–17). Furthermore, LPS is a known bacterial PAMP able to activate expression of inflammatory genes such as IL-1β, IL-8, IL-17A/F, and TNF-α in fishes (14, 39). Such actions are likely to be mediated by PRRs rather than TLR4, which is a known PRR in mammals, since the fish TLR4 homologs have much weaker binding affinities with LPS (40). However, the involvement of TLR4 as an associated PRR of LPS cannot be fully excluded. More recently, caspase 3 was identified as an intracellular PRR for recognizing LPS in mammals and is also present in fish (41). However, whether caspase 3 plays a role in LPS-induced IL-22 expression in fish remains to be investigated. Interestingly, IL-22 expression was inhibited at 24 h and 48 h after stimulation by PHA (Figure 3). In turbot, IL-22 was upregulated in the primary leukocytes of the head kidney and spleen at 3 and 6 h post-stimulation with PHA, but this was not the case at 24 h (14). These studies suggest that induction of IL-22 expression by PHA appears to be swift and transient.

The effects of several cytokines on IL-22 expression were also examined. IL-22 expression was upregulated by IL-2 and IL-34 in the primary head kidney leukocytes while it was inhibited by IL-4/13B and IL-10 (Figure 4). High amounts of IL-22 can be produced in lymphocytes involved in innate and adaptive immunity, including CD4 T cells, γδ T cells, NK cells, and innate lymphoid cells, and can be regulated by multiple factors (42, 43). Several cytokines such as IL-1β, IL-6, IL-23, and TGF-β1 are required for the development of Th17 cells which secrete IL-22 upon T cell receptor activation (42). IL-34 is known to regulate macrophage functions in mammals via the macrophage colony-stimulating receptor (MCSFR) (44). In our previous study, we demonstrated that recombinant IL-34 could upregulate the expression of proinflammatory cytokines such as IL-1β, IL-6, and IL-8 in enriched macrophage populations (25). In the present study, our data suggest that IL-22 may be produced by activated macrophages in fish and we consider that this warrants further investigation. The findings indicating the suppression of IL-22 expression by IL-4/13B and IL-10 in fish are also in agreement with past findings in mammals (42). These studies have indicated that Th2 cytokines play an inhibitory role in the regulation of Th17 responses in fish. However, observations of upregulation of IL-22 by IL-2 are also interesting. In the mammalian system, IL-2 together with IFN-γ promotes Th1 responses while antagonizing Th2 responses (45). The functions of fish IL-2 are still under debate, since fish seem to lack the highly affinitive binding private receptor equivalent to IL-2Rα (CD25) and rather facilitate binding to a private receptor shared by IL-15 (46).

The IL-22-mediated responses to bacterial infection have well been documented in fish. A number of studies have consistently shown that IL-22 can be upregulated at mRNA levels by gram-negative and gram-positive bacterial pathogens (13, 15–18). In a recent study, induction of the expression of the IL-22 protein was also observed in the gills of trout post-infection with *A. salmonicida* (21). In line with previous studies, it was not surprising that in our study mRNA expression of *Ci*IL-22 was induced in the spleen and hindgut of grass carp when they were infected with *F. columnare* (Figure 2B), which is a bacterial pathogen known to cause inflammation in fish (27, 28). In addition, our results indicate that the *Ci*IL-22 expression was increased in the tissues of fish infected with GCRV (Figure 2C). These findings suggest that *Ci*IL-22 is involved in the immune response to viral infection. In mice, the IL-22 secreted by conventional NK cells can reduce exacerbated inflammation caused by influenza virus infections and is required for the regeneration of damaged tracheal epithelial layers (47).

The biological effects of *Ci*IL-22 upon immune-related gene expression were evaluated in the primary head kidney leukocytes and CIK cells (a non-leukocyte cell line derived from grass carp kidney) (Figure 5). Our findings indicate that induction of a panel of cytokines including IL-1β, IL-6, IL-8, IL-10, IL-21, IL-22, IL-34, and TGF-β1 was pronounced in head kidney leukocytes but to a much lesser degree in CIK cells. These findings suggest that IL-22 may primarily target leukocytes. The differences may result from different numbers of IL-22 receptors expressed upon these cells. Interestingly, stimulatory effects were seen only in the leukocytes post-stimulation with low doses of IL-22 (2 and 20 ng/ml) while 200 ng/ml of IL-22 led to the inhibition of gene expression. Therefore, it is possible that the actions of IL-22 are dose dependent and could be regulated by a type of negative feedback. Moreover, the findings in the present study also imply that IL-22 elicits cellular responses through activation of STAT3-mediated signaling as STAT3 expression was upregulated by IL-22 in both the head kidney leukocytes and CIK cells (Figure 5). In mammals, STAT3 has been shown to be the central transcription factor mediating IL-22 signal transduction (48).

The mRNA levels of IL-22 expression have been relatively well-studied in fish (12, 14, 17, 20, 23). However, the cells secreting IL-22 have not been investigated until recently. Using monoclonal antibodies against synthesized peptides, Hu et al. (20) found that in trout, IL-22-producing cells were significantly increased in the gills and blood 24 h after i.p. infection with *A. salmonicida* and *Y. ruckeri*. Further, the IL-22^+^ cells were confirmed as present in the gill lamellae and in the interbranchial region. The gill interbranchial region contains high numbers of T cells and is considered as a secondary lymphoid tissue in fish (49). Therefore, it is possible to speculate that the IL-22^+^ cells therein are likely to be of lymphoid origin (20). Fish gut is also an important mucosal tissue and contains a diffuse gut-associated lymphoid tissue (GALT), although morphologically and functionally different from that in mammals (50). In the present study, the IL-22^+^ cells were clearly detected in the inner wall of the hindgut of fish infected with *F. columnare* but were not detected in the hindgut of healthy fish (Figure 7). These findings indicate that the cells were involved in mounting antibacterial defenses in the gut. The IL-22^+^ cells could be activated locally in the GALT or could have migrated from other sites as higher numbers of IL-22^+^ cells were also found in the head kidney (Figure 7). In mammals, IL-22 is mainly secreted by activated Th17 cells and innate lymphoid cells (51).

In summary, an IL-22 homolog was identified in grass carp, an economically important aquaculture species in China. *In vitro* expression analyses revealed that it could be upregulated by LPS and cytokines such as IL-2 and IL-34 while it was inhibited by Th2 cytokines. Bacterial and viral infections resulted in increases in IL-22 expression in tissues. The recombinant IL-22 was effective in inducing the expression of a number of inflammatory cytokines and STAT3 in the primary head kidney leukocytes. Lastly, the IL-22-producing cells were located in the inner walls of the hindgut and in the tubules of the head kidney. This highlights the involvement of IL-22 in regulating mucosal and systemic immunity and its critical roles in the clearance of infectious pathogens.

## Data Availability Statement

The datasets presented in this study can be found in online repositories. The names of the repository/repositories and accession number(s) can be found below: https://www.ncbi.nlm.nih.gov/genbank/, MN643172.

## Ethics Statement

The animal study was reviewed and approved by the ethics committee of laboratory animals of Shanghai Ocean University.

## Author Contributions

JZ conceived and designed the study. YY performed most of the experiments. JW, JX, QL, ZW, and XZ assisted in protein preparation and analyses. XA, QG, and XC provided bioinformatics assistance and support. JZ and YY wrote the manuscript. All authors approved the manuscript.

## Conflict of Interest

The authors declare that the research was conducted in the absence of any commercial or financial relationships that could be construed as a potential conflict of interest.
